# Supplementary material for: Variation in Neotropical river otter (Lontra longicaudis) diet: Effects of an invasive prey species
Source: PLoS One. 2019 Oct 3;14(10):e0217727. doi: 10.1371/journal.pone.0217727 (PMC6776311; doi:10.1371/journal.pone.0217727)
Supplement: S1 Table — (DOCX) [file pone.0217727.s001.docx]

**Supplementary Table 1.** Potential prey of Neotropical river otters found in basins sampled during this study: Usumacinta (U) and Mopan (M). The Usumacinta basin includes Pasion and San Pedro rivers. Fish species that have a maximum reported total length ≥ 10 cm were considered as potential prey. The maximum total length (MTL) of each species as well as its fractional trophic level index (FTL) and standard errors (SE) are from Froese and Pauly (2016). Scientific names follow Eschmeyer and Fong (2016).

| Species | | Basin | MTL | | FTL | | SE |
| --- | --- | --- | --- | --- | --- | --- | --- |
| Anguillidae |  |  |  | |  | |  |
| *Anguilla rostrata* (Lesueur 1817) | | U | 153 | | 3.8 | | 0.2 |
| Ariidae |  |  |  | |  | |  |
| *Bagre marinus* (Mitchill 1815) | | M | 69 | | 3.5 | | 0.5 |
| *Cathorops aguadulce* (Meek 1904) | | UM | 22.7 | | 4.4 | | 0.8 |
| *Potamarius nelsoni* (Evermann & Goldsborough 1902) | | U | 39 | | 3.6 | | 0.5 |
| *Sciades assimilis* (Günther 1864) | | M | 35 | | 3.6 | | 0.5 |
| Batrachoididae |  |  |  | |  | |  |
| *Batrachoides gilberti* Meek & Hildebrand 1928 | | M | 23 | | 3.7 | | 0.6 |
| *Batrachoides goldmani*Evermann & Goldsborough 1902 | | U | 21.6 | | 3.5 | | 0.6 |
| Belonidae |  |  |  | |  | |  |
| *Strongylura hubbsi* Collette 1974 | | U | 49.8 | | 4.1 | | 0.7 |
| *Strongylura marina* (Walbaum 1792) | | M | 111 | | 3 | | 0 |
| *Strongylura notata* (Poey 1860) | | M | 61 | | 4.4 | | 0.7 |
| Bryconidae |  |  |  | |  | |  |
| *Brycon guatemalensis* Regan 1908 | | UM | 59 | | 2.3 | | 0.3 |
| Carangidae |  |  |  | |  | |  |
| *Caranx latus* Agassiz 1831 | | M | 101 | | 4.2 | | 0.4 |
| *Oligoplites saurus* (Bloch & Schneider 1801) | | M | 35 | | 4.3 | | 0.5 |
| Centropomidae |  |  |  | |  | |  |
| *Centropomus ensiferus* Poey 1860 | | M | 36.2 | | 4.2 | | 0.6 |
| *Centropomus parallelus* Poey 1860 | | M | 72 | | 4.2 | | 0.7 |
| *Centropomus pectinatus* Poey 1860 | | M | 56 | | 4 | | 0.7 |
| *Centropomus undecimalis* (Bloch 1792) | | UM | 140 | | 4.2 | | 0.6 |
| Characidae |  |  |  | |  | |  |
| *Astyanax fasciatus* (Cuvier 1819) | | M | 16.8 | | 3 | | 0.3 |
| Cichlidae |  |  |  | |  | |  |
| *Chuco godmanni* (Günther 1862) | | M | 30 | | 2.7 | | 0.3 |
| *Chuco intermedium* (Günther 1862) | | UM | 20 | | 2.7 | | 0.3 |
| *Cincelichthys pearsei* (Hubbs 1936) | | U | 20 | | 2 | | 0 |
| *Cribroheros robertsoni* (Regan 1905) | | UM | 19 | | 3.4 | | 0.4 |
| *Cryptoheros cutteri* (Fowler 1932) | | M | 11.2 | |  | |  |
| *Cryptoheros spilurus* (Günther 1862) | | M | 12 | | 2 | | 0 |
| S1 Table. Continued | |  |  |  | |  | |
| Species | | Basin | MTL | FTL | | SE | |
| *Kihnichthys ufermanni*(Allgayer 2002) | | U | 25 | | 3.6 | | 0.5 |
| *Maskaheros argenteus*(Allgayer 1991) | | U | 27 | |  | |  |
| *Mayaheros alborus* (Hubbs 1936) | | U | 39.4 | | 3.9 | | 0.6 |
| *Mayaheros urophthalmus* (Günther 1862) | | M | 39.4 | | 3.9 | | 0.6 |
| *Oreochromis aureus* (Steindachner 1864) | | U | 45.7 | | 2.1 | | 0 |
| *Oscura heterospila* (Hubbs 1936) | | U | 24 | | 2.7 | | 0.3 |
| *Parachromis friedrichsthalii* (Heckel 1840) | | UM | 28 | | 4.2 | | 0.7 |
| *Parachromis managuensis* (Günther 1867) | | M | 55 | | 4 | | 0.6 |
| *Petenia splendida* Günther 1862 | | UM | 50 | | 4.5 | | 0.8 |
| *Rheoheros lentiginosus* (Steindachner 1864) | | U | 25 | | 2.8 | | 0.3 |
| *Rocio octofasciata* (Regan 1903) | | UM | 25 | | 3.5 | | 0.5 |
| *Thorichthys affinis* (Günther 1862) | | ? | 14 | | 3.2 | | 0.5 |
| *Thorichthys aureus* (Günther 1862) | | M | 15 | | 3.2 | | 0.5 |
| *Thorichthys helleri* (Steindachner 1864) | | U | 14.5 | | 3.2 | | 0.5 |
| *Thorichthys meeki* Brind 1918 | | UM | 17 | | 2 | | 0 |
| *Thorichthys pasionis* (Rivas 1962) | | U | 17 | | 3.2 | | 0.5 |
| *Trichromis salvini* (Günther 1862) | | UM | 22 | | 3.7 | | 0.52 |
| *Vieja bifasciata* (Steindachner 1864) | | U | 30 | | 2 | | 0 |
| *Vieja melanura* (Günther 1862) | | UM | 19 | | 2.6 | | 0.3 |
| Clupeidae |  |  |  | |  | |  |
| *Dorosoma anale* Meek 1904 | | UM | 20 | | 3.4 | | 0.4 |
| *Dorosoma petenense* (Günther 1867) | | UM | 33 | | 2.8 | | 0.1 |
| *Harengula jaguana* Poey 1865 | | M | 21.2 | | 3.4 | | 0 |
| *Opisthonema oglinum* (Lesueur 1818) | | M | 38 | | 4.5 | | 0 |
| Cyprinidae |  |  |  | |  | |  |
| *Ctenopharyngodon idella* (Valenciennes 1844) | | U | 150 | | 2 | | 0 |
| Cyprinodontidae |  |  |  | |  | |  |
| *Floridichthys polyommus* Hubbs 1936 | | M | 11 | | 2.9 | | 0.4 |
| Eleotridae |  |  |  | |  | |  |
| *Dormitator maculatus* (Bloch 1792) | | M | 70 | | 2.5 | | 1 |
| *Erotelis smaragdus* (Valenciennes 1837) | | M | 20 | | 3.4 | | 0.1 |
| *Gobiomorus dormitor* Lacepède 1800 | | UM | 90 | | 3.6 | | 0.1 |
| Gerreidae |  |  |  | |  | |  |
| *Diapterus rhombeus* (Cuvier 1829) | | M | 40 | | 3 | | 0.2 |
| *Eucinostomus gula* (Quoy & Gaimard 1824) | | M | 23 | | 2.7 | | 0.1 |
| *Eucinostomus melanopterus* (Bleeker 1863) | | M | 30 | | 3.4 | | 0.5 |
| *Eugerres brasilianus* (Cuvier 1830) | | M | 50 | | 3.4 | | 0.4 |
| *Eugerres mexicanus* (Steindachner 1863) | | U | 21.6 | | 3.4 | | 0.5 |
| *Eugerres plumieri* (Cuvier 1830) | | M | 40 | | 2.2 | | 0 |
| *Gerres cinereus* (Walbaum 1792) | | M | 20 | | 3.5 | | 0.2 |
| S1 Table. Continued | |  |  |  | |  | |
| Species | | Basin | MTL | FTL | | SE | |
| Gobiidae |  |  |  | |  | |  |
| *Evorthodus lyricus* (Girard 1858) | | M | 15 | | 3.4 | | 0.4 |
| *Gobioides broussonnetii* Lacepède 1800 | | M | 55.3 | | 3.7 | | 0.5 |
| Hemiramphidae |  |  |  | |  | |  |
| *Hyporhamphus mexicanus* Álvarez 1959 | | U | 16.1 | | 3 | | 0.4 |
| *Hyporhamphus roberti* (Valenciennes 1847) | | M | 32 | | 3 | | 0.4 |
| *Hyporhamphus unifasciatus* (Ranzani 1841) | | M | 30 | | 2 | | 0 |
| Heptapteridae |  |  |  | |  | |  |
| *Rhamdia guatemalensis* (Günther 1864) | | UM | 47.4 | | 3.9 | | 0.3 |
| *Rhamdia laticauda* (Kner 1858) | | UM | 22.5 | | 3.6 | | 0.4 |
| *Rhamdia quelen*(Quoy & Gaimard 1824) | | UM | 47.4 | | 9.9 | | 0.3 |
| Ictaluridae |  |  |  | |  | |  |
| *Ictalurus furcatus* (Valenciennes 1840) | | U | 165 | | 3.4 | | 0.4 |
| *Ictalurus meridionalis* (Günther 1864) | | U | 73 | | 3.7 | | 0.4 |
| Lacantuniidae |  |  |  | |  | |  |
| *Lacantunia enigmatica* Rodiles-Hernández, Hendrickson & Lundberg 2005 | | U | 42.7 | | 3.3 | | 0.5 |
| Lepisosteidae |  |  |  | |  | |  |
| *Atractosteus tropicus* Gill 1863 | | U | 125 | | 4.2 | | 0.7 |
| Lobotidae |  |  |  | |  | |  |
| *Lobotes surinamensis* (Bloch 1790) | | M | 110 | | 4 | | 0.5 |
| Loricariidae |  |  |  | |  | |  |
| *Pterygoplichthys disjunctivus* (Weber 1991) | | U | 70 | |  | |  |
| *Pterygoplichthys pardalis* (Castelnau 1855) | | U | 42.3 | | 2 | | 0 |
| Lutjanidae |  |  |  | |  | |  |
| *Lutjanus griseus* (Linnaeus 1758) | | M | 89 | | 4.2 | | 0.3 |
| *Lutjanus jocu* (Bloch & Schneider 1801) | | M | 128 | | 4.4 | | 0.3 |
| Megalopidae |  |  |  | |  | |  |
| *Megalops atlanticus* Valenciennes 1847 | | UM | 250 | | 4.5 | | 0 |
| Mugilidae |  |  |  | |  | |  |
| *Mugil cephalus* Linnaeus 1758 | | U | 100 | | 2.5 | | 0.2 |
| *Mugil curema* Valenciennes 1836 | | UM | 90 | | 2 | | 0 |
| *Mugil liza* Valenciennes 1836 | | M | 80 | | 2 | | 0 |
| *Mugil trichodon* Poey 1875 | | M | 46 | | 2 | | 0 |
| Poeciliidae |  |  |  | |  | |  |
| *Belonesox belizanus*Kner 1860 | | UM | 20 | | 2.9 | | 0.4 |
| *Poecilia latipinna* (Lesueur 1821) | | M | 15 | | 2 | | 0 |
| *Poecilia mexicana* Steindachner 1863 | | UM | 11 | | 2 | | 0 |
| *Xiphophorus hellerii* Heckel 1848 | | UM | 14 | | 3.2 | | 0.4 |
| S1 Table. Continued | |  |  |  | |  | |
| Species | | Basin | MTL | FTL | | SE | |
| Sciaenidae |  |  |  | |  | |  |
| *Aplodinotus grunniens* Rafinesque 1819 | | U | 95 | | 3.4 | | 0.4 |
| Sparidae |  |  |  | |  | |  |
| *Archosargus aries* (Valenciennes 1830) | | M | 91 | | 3.5 | | 0.5 |
| Synbranchidae |  |  |  | |  | |  |
| *Ophisternon aenigmaticum* Rosen & Greenwood 1976 | | UM | 80 | | 3.3 | | 3 |
| *Synbranchus marmoratus* Bloch 1795 | | M | 150 | | 2.8 | | 0.5 |
